# *QuickStats*: Percentage* of Children and Teens Aged 4–17 Years Ever Diagnosed with Attention-Deficit/Hyperactivity Disorder (ADHD),^†^ by Sex and Urbanization^§^ of County of Residence — National Health Interview Survey,^¶^ 2013–2015

**DOI:** 10.15585/mmwr.mm6623a7

**Published:** 2017-06-16

**Figure:**
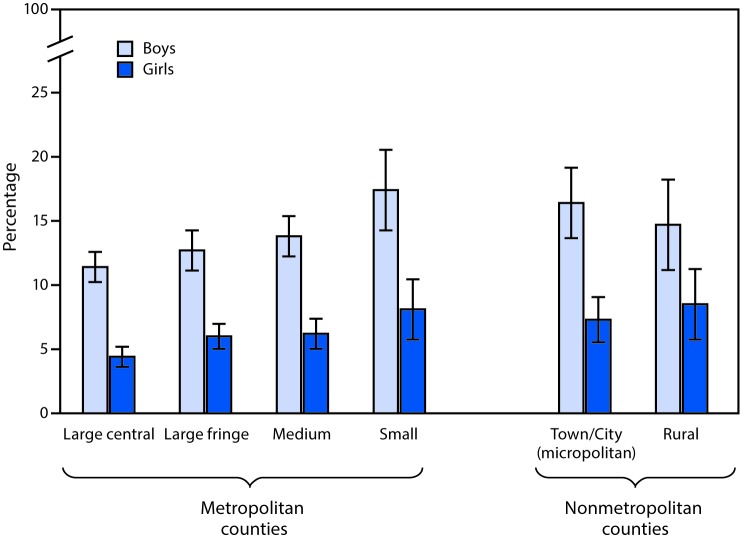
During 2013−2015, the percentage of children and teens aged 4–17 years who had ever received a diagnosis of ADHD was significantly higher among boys than among girls within all urbanization levels. Among boys, those living in small metro and nonmetro micropolitan areas were more likely to have received a diagnosis of ADHD (17.4% and 16.4%, respectively) than were those living in large central (11.4%) and large fringe (12.7%) metropolitan areas. Among girls, those living in large central areas were less likely to have received a diagnosis of ADHD (4.4%) than those living in each of the other five types of urban/rural areas.

**Source:** National Center for Health Statistics. National Health Interview Survey, 2013–2015. https://www.cdc.gov/nchs/nhis.htm.

